# Does self-regulation and autonomic regulation have an influence on survival in breast and colon carcinoma patients? results of a prospective outcome study

**DOI:** 10.1186/1477-7525-9-85

**Published:** 2011-09-30

**Authors:** Matthias Kröz, Marcus Reif, Arndt Büssing, Roland Zerm, Gene Feder, Angelina Bockelbrink, Hans Broder von Laue, Harald Matthes H, Stefan N Willich, Matthias Girke

**Affiliations:** 1 Research Institute Havelhöhe (FIH), Gemeinschaftskrankenhaus Havelhöhe, Kladower Damm 221, 14089 Berlin, Germany; 2 Department of Internal Medicine, Gemeinschaftskrankenhaus Havelhöhe, Kladower Damm 221, 14089 Berlin, Germany; 3 Institute for Social Medicine and Epidemiology, and Health Economics, Charité CCM, 10098 Berlin, Germany; 4 Institute for Clinical Research (IKF), Hardenbergstr. 19, D-10623 Berlin, Germany; 5 Center of Integrative Medicine, Professorship Quality of Life, Spirituality and Coping, University of Witten/Herdecke, Gerhard-Kienle-Weg 4, 58313 Herdecke, Germany; 6 Academic Unit of Primary Care, School of Social and Community Medicine, University of Bristol, 25 Belgrave Road, London BS8 2AA, UK; 7 Humanus Institute, Kladower Damm 221, 14089 Berlin, Germany

**Keywords:** Autonomic regulation (aR), breast cancer, colorectal cancer, coping, self-regulation (SR)

## Abstract

**Background:**

Cancer Related Fatigue (CRF) and circadian rhythm have a great impact on the quality of life (HRQL) of patients with breast (BC) and colon cancer (CRC). Other patient related outcomes in oncology are measured by new instruments focusing on adaptive characteristics such as sense of coherence or self-regulation, which could be more appropriate as a prognostic tool than classical HRQL. The aim of this study was to assess the association of autonomic regulation (aR) and self-regulation (SR) with survival.

**Methods:**

146 cancer patients and 120 healthy controls took part in an initial evaluation in 2000/2001. At a median follow up of 5.9 years later, 62 of 95 BC, 17 of 51 CRC patients, and 85 of 117 healthy controls took part in the follow-up study. 41 participants had died. For the follow-up evaluation, participants were requested to complete the standardized aR and SR questionnaires.

**Results:**

On average, cancer patients had survived for 10.1 years with the disease. Using a Cox proportional hazard regression with stepwise variables such as age, diagnosis group, Charlson co-morbidity index, body mass index (BMI)) aR and SR. SR were identified as independent parameters with potential prognostic relevance on survival While aR did not significantly influence survival, SR showed a positive and independent impact on survival (OR = 0.589; 95%-CI: 0.354 - 0.979). This positive effect persisted significantly in the sensitivity analysis of the subgroup of tumour patients and in the subscale 'Achieve satisfaction and well-being' and by tendency in the UICC stages nested for the different diagnoses groups.

**Conclusions:**

Self-regulation might be an independent prognostic factor for the survival of breast and colon carcinoma patients and merits further prospective studies.

## Background

Cancer Related Fatigue (CRF) is one of the most common symptoms experienced by cancer patients receiving palliative care [1] and patients treated with chemo- or radiotherapy [2]; it is also relatively common in disease-free cancer patients. In a British study 58% of all oncology outpatients reported that fatigue affected them 'somewhat or very much' and described it as the most important symptom which is often not being well-managed [3].

CRF is often associated with sleep disturbances. From the 31% of all cancer patients suffering from insomnia in a large cross sectional study, 76% reported disturbed sleep continuation [4] Disturbed rest/activity and affected circadian rhythms may aggravate CRF and depressive symptoms in adjuvant treated breast cancer patients [5] and diminishes health-related quality of life (HRQL) in breast [5] and colorectal cancer patients [6]. In metastasized colon carcinoma patients actimetrically measured disturbed rest/activity rhythm is associated with shorter survival [7] and in breast cancer patients (BC) diminished circadian cortisol rhythm is associated with higher mortality [8]. Beside physiological measures, another epidemiological available approach is measuring rest/activity regulation with a validated assessment applicable in clinical settings as a part of a questionnaire measuring different functions (1. rest/activity, 2. orthostatic-circulatory, 3. digestion) of autonomic regulation (aR), which to our knowledge is the first scale measuring autonomic functioning with sufficient validity [9].

There is some evidence that questionnaires measuring patients' adaptive capacity towards disease and health-orientated life-style change, such as the 'sense of coherence' (SOC) [10] or 'self-regulation' (SR) [11], could have stronger association with prognosis in oncology or other chronic conditions than HRQL scales [12-15]. One of these tools is based on Antonovsky's core question 'What may keep one healthy?' For Antonovsky, SOC is based on three components which are prerequisites for salutogenesis, i.e., comprehensibility, meaningfulness, and manageability [10]. Up to now, inventories which capture the SOC based on Antonovsky's concept of salutogenesis are predominantly validated for patients with psychosomatic or mental health conditions, psychiatric patients. Moreover, they are often used in sociological studies as a stable personality trait marker, while they have not been developed as clinical measures for physical and oncological conditions [10,16-18].

Another scale based on salutogenesis with a clinical application is the psychosomatic Self-Regulation Scale (SR) developed by Grossarth-Maticek. This questionnaire deals with the "ability to actively achieve well-being, inner equilibrium, appropriate stimulation, a feeling of competence, and a sense of being able to control stressful situations" [19]. Grossarth-Maticek & Eysenck characterized this concept as a short-hand personality trait term which "covers a conglomerate of concepts" related to reaction to a variety of stressors and coping mechanisms and not only as 'locus of control' [15]. The SR scale has been developed as an epidemiological, preventive health care and clinical measure in a long and short version, and has been validated, applied and evaluated against physical risk factors prospectively in breast and colorectal cancer patients [11,14]. SR short version is capturing two factors: 1) ability to 'change behaviour to reach a goal' and 2) a subscale called 'Achieve satisfaction and well-being' [20].

The aim of our study was to assess the influence on overall survival of

1) the validated autonomic regulation scale (aR) (and its subscale for rest/activity rhythm (R/A.aR)) [9] and of

2) the short version of the psychosomatic Self-Regulation Scale (SR) (and its subscales 'Change behaviour to reach goal' and 'Achieve satisfaction and well-being') [20].

## Methods

### Patients

This multicenter observational study was conducted at the Department of Internal Medicine, Surgery and Gynaecology of the Havelhöhe Community Hospital, Berlin, the Öschelbronn Oncological Practice and the Wuppertal Endocrinology Practice from April 2000 - November 2001. The participants of the study consisted of healthy volunteers and in total seven groups of patients. The latter were recruited consecutively among inpatients at the Havelhöhe Hospital and from outpatients in the two practises. In this paper we report the results from the breast cancer and colorectal cancer group and the healthy controls.

The inclusion criterion was histologically proven breast or colorectal cancer. The control group was recruited from the Havelhöhe Hospital staff and their relatives. Exclusion criteria were other severe organic diseases, manifest psychosis, severe immobilisation or a Karnofsky index (KPI) < 50%, uncontrolled pain, recent operations (< 1 week prior to study recruitment) and recent chemo- or radiotherapy (< 3 weeks prior recruitment). Among 131 healthy volunteers, 95 breast cancer (all female) patients and 51 colorectal cancer patients (30 female/58.8%), all cancer patients and 120 healthy controls (80 female/66.7%) (C) gave their written consent and took part in an initial evaluation in 2000/2001 (table 1). According to our institutional standard in 2000, we did not ask ethical approval in anonymous questionnaire based observational studies.

**Table 1:** Sociodemographic data of the study groups including stage, therapies, participation rate

	in 2000-2001				in 2006-2007		
	
	CG	BC	CRC		CG	BC	CRC
				Died	2	14	25
Invited (n):	131	95	51		117	81	26
Consented (n):	120	95	51		85	62	17
Complete Data (n):	115	95	49				
Censored Data					7	4	1
Women (n):	80	95	30				
Age (mean):	54	57.1	62				
(SD)	(14.2)	(9.9)	(12.2)				
**Marital Status:**							
Married (n/%)	75/65.2	59/62.1	33/67.3				
Single (n/%)	13/11.3	8/8.4	6/12.2				
Divorced (n/%)	15/13.0	13/13.7	5/10.2				
Widowed (n/%)	8/7.0	9/9.5	4/8.2				
No details available (n/%)	4/3.4	6/6.3	1/2.0				
**Most recent profession:**							
Worker (n/%)	6/5.2	12/12.6	12/24.4				
Employee/civil servant (n/%)	72/62.6	45/47.4	22/44.9				
Self employed (n/%)	19/16.5	8/8.4	5/10.2				
House wife/husband (n/%)	12/10.4	23/24.2	10/20.4				
Still in education (n/%)	3/2.6	0/0	0/0				
No details available (n/%)	3/2.6	7/7.3	0/0				
Pension (n/%)							
**Karnofsky-I**.	%(SD) of survivors:	96.7(7.2)	88.2(12.5)				
**UICC stages (n/%):**							
I		28/29.5	6/12				
II		37/38.9	9/18				
I/II		3/3.2	-				
III		4/4.2	15/29				
II/III		-	2/4				
IV		23/24.2	19/37				
**Grading (SD):**		2.0(0.62)	2.25(0.51)				
**Metastasis localisation (n/%):**							
Bone		5/5.2	1/1.9				
Liver		-	8/15.7				
Peritoneal		-	2/3.9				
Lung		2/2.1	-				
Multiple		9/9.4	7/13.7				
others		5/5.2	1/1.9				
**Duration of disease**	(Mean/SD):	4.7/5.6	1.7/2.3				
**Menopausal status at diagnosis:**							
Premenopausal (n/%)		38/39.6					
Postmenopausal (n/%)		53/55.2					
**Treatment:**							
Operation: n/%		93/97.9	51/100				
Chemotherapy: n/%		55/57.9	22/44				
Radiotherapy: n/%		55/57.9	8/15.7				
antihormonal therapy.: n/%		55/57.9	-				
mistletoe therapy: n/%		79/83.2	38/71.7				

Abbreviations: control group (CG), breast cancer (BC), colorectal cancer (CRC)

**Figure 1:**
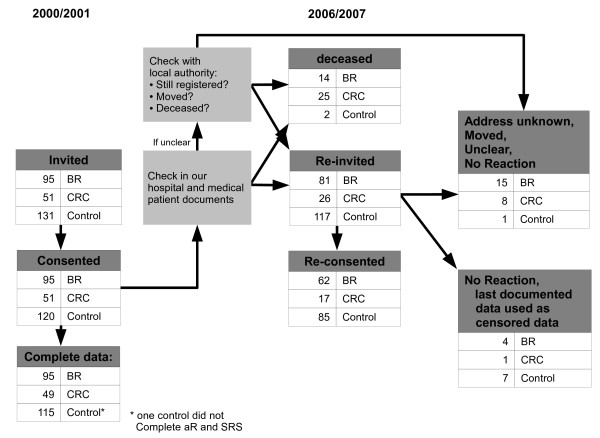
**Flow chart of participants recruiting 2000/2001 and 2006/2007**.

From April 2006 to October 2007 we conducted a re-assessment of all participants of the 2000-2001 study. After checking our medical patients documents we checked than if participants were still registered with the local administration; if they were no longer registered we investigated whether they had died (registered death date) or moved. (Figure 1, table 1).

### Measures

Participants were given the aR-scale (table 2), the SR scale (table 3) and the Hospital Anxiety and Depression Scale (HADS) [21] in 2000/2001 and 2006/2007, and a self-completion version of the Karnofsky index (KPI).

**Table 2:** Items on autonomic regulation

Questions autonomic regulation	Possible answers On autonomic regulation		
	Low = 1	average = 2	high = 3
**Orthostatic-circulatory regulation**			

Do you suffer from dizzy spells?	frequently	occasionally	never

Do you suffer from dizziness when you look down from a height?	frequently	occasionally	never

Do you suffer from dizziness when you get up in the morning?	frequently	occasionally	never

Do you suffer from dizziness when you straighten up or bend down?	frequently	occasionally	never

Do you tend to have cold or cold-sweaty hands even in the warmer months?	frequently	occasionally	rarely

Do you suffer from travel sickness (e.g. sea sickness)?	frequently	occasionally	almost

Do you get dizzy from circular motions (when on a roundabout, for example)?	frequently	occasionally	almost

**Rest/activity regulation**			

Do you have to pull yourself together to go to work?	frequently	occasionally	rarely

Do you feel rested in the morning	rarely	occasionally	frequently

Do you have problems falling asleep?	frequently	occasionally	rarely

Do you tend to sweat?	frequently	occasionally	rarely

Do you suffer from disturbed sleep?	frequently	occasionally	rarely

At what time of the day do you feel most comfortable?	evening	in the middle of the day	morning

Do you tend to sweat at night?	frequently	occasionally	rarely

Do you tend to have stomach growling?	frequently	occasionally	rarely

**Digestive regulation**			

How often do you have bowel movements?	< 1/day	approx. 1/day	> 1/day

Do you normally have bowel movements at regular times?	rarely	occasionally	frequently

Do you suffer from constipation?	frequently	occasionally	rarely

**18 item sum scale**			

18 validated items on autonomic regulation with the three subscales orthostatic-circulatory, rest/activity and digestive regulation, including the individual, possible answers. The left answer corresponds to low (1 point), the middle to average (2 points) and the right to high autonomic regulation (3 points).

**Table 3:** Items of the self-regulation questionnaire with the two subscales 1) Ability to Change Behaviour in Order to Reach Goals and 2) Achieve Satisfaction and Well-Being

	Questionnaire on Self-regulation
	**1. Ability to Change Behaviour in Order to Reach Goals**

SR12	Ability for new behaviour pattern

SR11	Ability to change behaviour to reach pleasant outcome

SR6	Threatening situations: behave to emerge safe

SR10	Find standpoints/behaviour pattern which allow pleasant problem solving

SR7	Attain most important objectives

SR9	Disappointment: no reason for resignation, but cause to change behaviour

SR13	Because of behaviour desired proximity and required distance to important others

SR4	Expand various activities until states change to total satisfaction

	**2. Achieve Satisfaction and Well-Being**

SR15	Achieve well-being by daily activities

SR14	Activate inner satisfaction over and over again by daily activities

SR2	Actualize wishes and satisfy needs

SR5	arrange different areas of life optimal so that well-being can result

SR3	Achieve situations/states which restore well-being

SR1	Achieve situations/states which motivate

SR8	Achieve situations/states which satisfy wishes and needs optimal

SR16	Behaviour gives rise to situations which cause experiences full of relish

1) The **autonomic regulation **(aR) scale addresses the state of regulation of different autonomic functions. The 18-item scale measures the three factor model Orthostatic-Circulatory, Rest/Activity and Digestive regulation with a three-point Likert scale and has a satisfying internal consistency (Cronbach-α: rα = 0.65-0.75), and satisfying to good test-retest reliability (rrt = 0.70 - 85), and good validity [9].

2) The **short questionnaire on self-regulation **(SR) is a scale with 16 items to measure one's activity towards harmonizing and health orientation with a six-point Likert scale ranging from 1 (very weak) to 6 (very strong) (addition of the 16 items and division by 16: Range 1-6. The questionnaire consists of two subscales with eight items each: 1) 'Change behaviour to reach goal' and 2) 'Achieve satisfaction and well-being'. Higher scoring indicates better self-regulation. The self-regulation questionnaire is highly reliable and valid with a good - very good internal consistency (Cronbach-α: rα = 0.80-0.95) and satisfying - good test-retest reliability = 0.73-0.82) [11,20].

3) The **Karnofsky performance index **(KPI) is a commonly used functional measure for oncology patients [22]. Although it was designed for clinical assessment by physicians, its categorization is easy to understand for patients as well and was thus be used for a patient-based evaluation.

4) The German version of the '**Hospital Anxiety and Depression Scale**' (HADS-D) consists of 14 items (7 for anxiety and 7 for depression) with a four-point Likert scale (0-21 for both). Higher scoring indicates more symptoms. The HADS is highly reliable and valid and is an extensively used scale in internal medicine research [21].

5) The **Charlson co-morbidity index **is an often used index in internal medicine and oncology for co-morbidity with a robust correlation with outcome [23].

### Statistical analysis

Analysis was performed with SPSS 16.0 and SAS 9.1.3 software packages. Relevant factors influencing survival were identified by a variable selection procedure using Cox proportional hazard regression. Parameters included in the selection process as independent factors included diagnostic groups, age, sex, Charlson co-morbidity index, nicotine abuse, body mass index (BMI), anxiety and depression scores of the HADS, allergy and marital status, aR and SRS. Primary variable selection was a stepwise selection procedure, a combination of forward and backward variable selection. This procedure computed the score statistic for each effect not yet in the model. The parameter with the largest of these score statistics, when significant at an error level of 0.25, was added to the model. Any parameter could again be removed from the selected variables model if its p-value increased over a threshold of 0.15 after inclusion or removal of other parameters. The outcome of the stepwise selection was compared with pure forward and backward selection techniques. All procedures resulted in the same parameters remaining in the model. This consistency in parameter selection was also the case in the sensitivity analyses.

The proportionality assumption of the selected model was checked by a resample Kolmogorov supremum test with 1000 simulation iterations as suggested by Lin et al. [24]. Here, age turned out to significantly deviate from proportionality assumptions. After graphical inspection, age was squared for inclusion. Thereby not only the non-proportionality of this parameter was resolved but the Cox model resulted in smaller p-values for all other parameters except for BMI.

Because of differences in prognosis between both cancer groups, stage according to Union Internationale Contre le Cancer (UICC), nested in the different diagnostic groups, was integrated in a sensitivity analysis (healthy subjects were allocated to UICC stage 0). Further sensitivity analyses regarded only the sub-group of tumor patients, with and without additionally including tumor and lymph node staging, presence of metastases, grading, and the use of chemo-, radio- or mistletoe therapy in the parameter selection process. An analysis aiming to include both UICC staging and tumor patient sub-sample failed to result in a reliable model estimate due to an insufficient number of events.

In order to illustrate the influence of SRS for all diagnosis groups, in a Kaplan-Meier survival plot we allocated all patients at a SRS of 3.85 (which is a clinical useful cut-off between moderate and good SRS) into a high SRS (> 3.85) or low SRS (< 3.85) class, respectively.

## Results

At study inclusion breast cancer patients participating in the study had a mean disease duration of 4.7 years, 13 (13.7%) of them a disease duration of less than 1 year, only 3 (3.2%) an operation between 2 and 4 weeks before. About half the participants were postmenopausal at diagnosis (55.2%) and 75.8% (4.2% in UICC 3) did not have metastatic disease stage. 97.9% had been operated and 57.9% of all had received standard radio-chemotherapy and were still receiving hormonal treatment (table 1). Colorectal cancer patients participating in the study had a mean disease duration of 1.7 years, 23 (45.1%) of them a disease duration of less than 1 year, 13 (25.5%) in the last month. Only 63% did not have metastatic disease (29% UICC 3). 44% had received chemotherapy and 15.7% radiotherapy (table 1). Both groups had a high rate of concomitant mistletoe therapy (83.2% and 71.7%) (table 1).

With a median follow up of 5.9 years, 62 of 81 breast cancer patients (BC), 17 of 26 colorectal cancer patients (CRC), and 85 of 117 controls (C) (in total 73.2%) participated in the follow-up study (equivalent to 61.6% of the initial sample). From the initial cohort, 41 of 266 participants (14 BC, 25 CRC, 2 C) had died (15.4%), with 77.1% of patients of the entire initial cohort (table 1) responding (table 1). Mean survival time of the cancer groups was 10.1 years (SD = 3.9). Mean age of the whole group was 60.2 years (SD = 12.2); for details of the study groups refer to table 1.

The Karnofsky performance index (KPI) of the cancer survivors was 96.7% (all 92.5%) in breast cancer and 88.2% in colorectal cancer (all 83.3%) at baseline. AR sum scale correlates with SR initially with r = 0.34.

There were three bivariate correlations within these variables above 0.5, with the highest value of 0.62 between the anxiety and the depression scale of the HADS and KPI with diagnosis and UICC stage (-0.53--0.61); thus, multi-collinearity was of no concern, as was confirmed by ridge analysis. Nevertheless, KPI was not integrated in the stepwise variable selection because of its moderate to strong correlation with diagnosis and UICC stage.

In the final model after variables selection the diagnosis groups colorectal carcinoma (HR = 23.515, CI = 5.183-106.683, p < 0.0001 and breast cancer (HR = 5.244, CI = 1.111-24.757, p = 0.0364), the Charlson co-morbidity index (HR = 1.389, CI = 1.043-1.848, p = 0.0245) and high self-regulation show positive and independent impact on survival, with an HR of 0.589 (95%-CI: 0.354-0.979) (table 4). This positive effect is corroborated by the analysis of the two subscales for 'Achieve satisfaction and well-being' (HR = 0.560; 95%-CI: 0.350-0.895) and by tendency for 'Change behaviour to reach goal' (HR = 0.663; 95%-CI: 0.413-1.066). On the other hand aR sum scale and rest/activity regulation subscale (R/A.aR) have no significant influence on survival (aR: HR = 1.069, CI = 0.992-1.152; R/A.aR: HR = 1.069, 0.948-1.205).

**Table 4:** Final model after variable selection for breast and colorectal cancer and control group, significant results are printed in bold

Parameter	DF	Parameter Estimate	Standard Error	Chi-Square	P value	Hazard-Ratio	95% Hazard Ratio Confidence Limits	
**Diagnosis-group** **Colon-CA**	**1**	**3.15763**	**0.77156**	**16.7488**	**< .0001**	**23.515**	**5.183**	**106.683**
**Diagnosis-group** **breast-CA**	**1**	**1.65702**	**0.79189**	**4.3785**	**0.0364**	**5.244**	**1.111**	**24.757**
Age, Hazard Rate/10 years	1	0.33779	0.18621	3.2906	0.0697	1.402	0.973	2.019
**Charlson Comorbidity Index**	**1**	**0.32824**	**0.14593**	**5.0593**	**0.0245**	**1.389**	**1.043**	**1.848**
BMI to day	1	-0.08717	0.05667	2.3658	0.1240	0.917	0.820	1.024

aR score	1	0.06639	0.03810	3.0368	0.0814	1.069	0.992	1.152
**Self-regulation-Score**	**1**	**-0.52945**	**0.25945**	**4.1645**	**0.0413**	**0.589**	**0.354**	**0.979**

We conducted a second stepwise variables selection limited to the two cancer groups with the above used candidates and included chemotherapy, radiotherapy, mistletoe therapy, metastases (yes/no), grading (1-3). In the final model entered the following candidates: diagnosis colorectal cancer (HR = 22.106, CI = 5.404-90.424), metastasis (HR = 25.954, CI = 7.558-89.128), grading (HR = 0.179, CI = 0.072-0.446), age (HR = 1.610, CI = 1.037-2.498) and self-regulation (HR = 0.426, CI = 0.184-0.985) (table 5).

**Table 5:** Final model after variable selection for breast and colorectal cancer, significant results are printed in bold

Analysis of Maximum Likelihood Estimates								
**Parameter**	**DF**	**Parameter** **Estimate**	**Standard** **Error**	**Chi-Square**	**p-value**	**Hazard** **Ratio**	**95% Hazard Ratio Confidence Limits**	

**Diagnosis-Group Colorectal-cancer**	**1**	**3.09583**	**0.71873**	**18.5535**	**< .0001**	**22.106**	**5.404**	**90.424**
**Age**	**1**	**0.47614**	**0.22422**	**4.5094**	**0.0337**	**1.610**	**1.037**	**2.498**
Body mass-index	1	-0.13025	0.07652	2.8972	0.0887	0.878	0.756	1.020
Trait aR-score	1	0.09607	0.05278	3.3127	0.0687	1.101	0.993	1.221
**Self-regulation-Score**	**1**	**-0.85285**	**0.42751**	**3.9797**	**0.0461**	**0.426**	**0.184**	**0.985**
**Grading**	**1**	**-1.71907**	**0.46490**	**13.6731**	**0.0002**	**0.179**	**0.072**	**0.446**
**Metastases**	**1**	**3.25634**	**0.62947**	**26.7612**	**< .0001**	**25.954**	**7.558**	**89.128**

**Figure 2:**
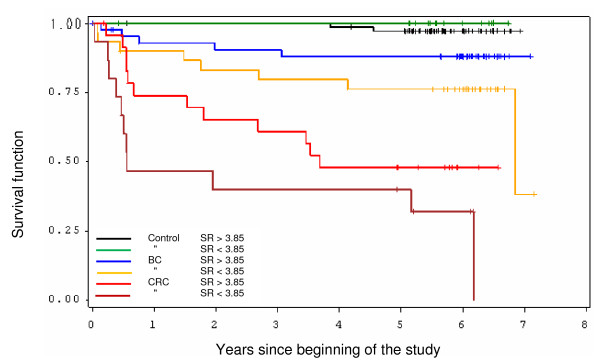
**The Kaplan-Meyer survival function was separately plotted for high and low self-regulation for control, breast cancer and colorectal cancer patients group**.

The sensitivity analysis, with nested UICC stages for both cancer groups, clearly resulted in a reduction in the parameters age and Charlson co-morbidity index, even if these variables were only moderately correlated with UICC stage (0.17 and 0.16, respectively). Estimates of SRS, on the other hand, were nearly unaffected in this model (HR = 0.565, 95%-CI: 0.306-1.045) but failed the 5% threshold (p = 0.0686) because of decrease sample number and consecutive increasing confidence interval (table 6). In the Kaplan Meier survival plot, colorectal cancer patients with low SR had the highest mortality, followed by the CRC-patients with high SR and breast cancer patients with low SR and high SR (Figure 2).

**Table 6:** Sensitivity analysis, final model after variable selection and UICC stages instead of diagnosis classes, significant results are plotted in bold

Parameter	stage	DF	Parameter Estimate	Standard Error	Chi-Square	P value	Hazard Ratio	95% Hazard Ratio Confidence Limits	
**Age (per 10 years)**		**1**	**0.36142**	**0.18408**	**3.8549**	**0.0496**	**1.435**	**1.001**	**2.059**
**Charlson index**		1	0.19521	0.13789	2.0042	0.1569	1.216	0.928	1.593
**BMI_to day**		1	-0.06812	0.05129	1.7636	0.1842	0.934	0.845	1.033
**aR score**		1	0.06235	0.04595	1.8414	0.1748	1.064	0.973	1.165
***Self-regulation score***		*1*	*-0.57008*	*0.31308*	*3.3155*	*0.0686*	*0.565*	*0.306*	*1.045*

**UICC CRC**	**1**	1	1.90827	1.24262	2.3583	0.1246	.		
**UICCCRC**	**2**	1	1.70462	1.24400	1.8777	0.1706	.		
**UICC CRC**	**3**	**1**	**2.78735**	**0.94298**	**8.7373**	**0.0031**	.		
**UICC CRC**	**4**	**1**	**5.27866**	**0.83612**	**39.8576**	**< .0001**	.		
**UICC BC**	**1**	1	0.38145	1.24018	0.0946	0.7584	.		
**UICC BC**	**2**	1	0.23170	1.19157	0.0378	0.8458	.		
**UICC BC**	**3**	**1**	**2.94979**	**1.28419**	**5.2763**	**0.0216**	.		
**UICC BC**	**4**	**1**	**4.11188**	**0.87876**	**21.8947**	**< .0001**	.		
**UICC control**	**0**	0	0	.	.	.	.		

## Discussion

In this study we found that, in addition to diagnostic group, UICC stages and the Charlson co-morbidity index, the self regulation (SR) scale (in particular its subscales 'Achieve satisfaction and well-being)' was a significant independent positive predictor of survival of breast and colorectal cancer patients. The autonomic regulation (aR) scale had no significant prognostic value.

Our findings on self-regulation are consistent with results of another research group that found that self-regulation is positively associated with patients survival with a range of solid tumours [11,19]. Self-Regulation (SR) is thought to represent the "ability to actively achieve well-being, inner equilibrium, appropriate stimulation and feeling of competence to control and manage stressful situations"[20] and shows repeatedly low-moderate correlations with aR (0.30-0.38) [25,20]. Unpublished data from our study group show a strong correlation between SR and the three SOC-subscales (comprehensibility, manageability and meaningfulness) from r = 0.70 to 0.73 (p < 0.05) which suggest that SOC/resilience might be connected with a goal-orientated change of lifestyle and orientation towards wellbeing. Gender specific coping strategies have been articulated, with women using a more emotion-based and men a more problem-orientated strategy. This distinction corresponds to the two subscales of self-regulation and the stronger relationship of "well-being orientation" to prognosis could be a function of our predominantly female sample [26]. Frentzel-Beyme & Grossarth hypothesized that highly self-regulated persons are more capable coping with sources of uncertainty and instability. The authors assume that people with well-regulated behaviour have a psycho-neuro-physiological basis for better competence and defence against health hazards [27]. The actual mechanism for the interaction of self-regulation and SOC with physiological processes remains unclear [28]. Both cross-sectional and prospective data show a positive association of the SOC scale to cancer survival and lower cancer incidence that are consistent with our results [13,29] if this depends on a higher resilience towards social stress, and a higher ability to adapt remains unclear [30]. However, this match with data from the self-regulation scale that autonomy helps for better stress management, less neuroticsm, better HRQL and initiative power and could be therefore helpful tool in preventive medicine [14,20].

Our findings support the case for developing interventions to improve self-regulation in cancer patients. Grossarth-Maticek & Eysenck propose autonomy training for the improvement of patients' self-regulation [31] and this has been tested in breast cancer prevention [32] with initial positive findings [15]. There is still a need for larger prospective observational studies alongside robust pragmatic trials of interventions based on the development of self-regulation. Although it has been reported that the application of mistletoe extracts may improve the self-regulation and survival of breast cancer and gynaecological cancer patients [33,34], in this study we did not find a significant influence of mistletoe extract application on self-regulation and survival, which were influenced by operation, chemo- and radiotherapy. This effect could be due to the high operation rate and mistletoe baseline application rate and the small sample size (compare table 1).

Colorectal cancer patients have in comparable stages with breast cancer patients an inferior survival which is banal news [35]. In our data CRC patients are more likely to be in stage III or IV with a relative low chemotherapy treatment frequency probably because of the strong complementary therapy desire of these patients in our centre for integrative medicine and a high mistletoe treatment rate etc. The breast cancer group consisted of more long term-survivors. In both cancer groups UICC stage and grading were strong prognostic factors alongside self-regulation. In cross-sectional studies low self-regulation was correlated with higher anxiety, depression and lower HRQL [20]. In a prospective study, multivariate analysis indicated that self-regulation can be a cofactor together with autonomic regulation for anxiety and an independent factor for depression. Hence, in conclusion, further studies are necessary to clarify if high self-regulation is an independent influencing factor, or is influenced due to the lack of anxiety, depression, demoralisation or risk factors. Thus, in the self-regulation concept we still have to deal with the same crucial question as for SOC, i.e., whether it is cause or effect [36].

Studies have measured the impact of disturbed rest/activity in metastasized colorectal cancer on survival [7,37] and HRQL [6]. According to meta-analysis, physical activity stabilizes not only daily activity and rest/activity rhythm but is actually the treatment with the highest evidence of improving cancer-related fatigue [38]. In large tertiary prevention studies it achieves intensity dependent a relative-risk reduction for colon and breast carcinoma until 50-57% [39,40]. In metastasized breast cancer, a reduced circadian cortisol rhythm is associated with higher mortality [8]. These results principally reflect two aspects: firstly the potential importance of disturbed circadian rhythm on survival, and secondly that disturbed and flattened cortisol rhythm is a distress marker with an influence on reduced HRQL, higher fatigue level [8] and higher prevalence of un-refreshing and disturbed sleep in breast cancer [41]. Even if there are differences in the frequency of insomnia between breast and colorectal cancer [41,42], there is a growing amount of basic research showing that a disturbed circadian rhythm could play an important role in malignant growth control in these and other cancers [43,44]. In spite of unclear underlying mechanisms, there is growing evidence that disturbed rest/activity and circadian rhythm are interrelated with CRF and sleep disturbances in both cancer groups [5,41,45]. CRF highly correlates with global HRQL and physical functioning [46] and in face of contradictory results fatigue, physical and emotional functioning in breast cancer and global health and particularly social functioning in colorectal cancer could be prognostic indictors of survival [47,48]. To clarify if and how strongly psychometrically measured rest/activity regulation is correlated with actigraphically measured rest/activity, we are actually conducting two ongoing studies. In a prospective study we determined that psychometrically measured autonomic regulation is significantly reducing cancer-related fatigue and cognitive fatigue [49]. However, the relevance of a disturbed rest/activity or circadian rhythm in metastasized cancer patients requires further research and is still unclear in non-metastasized cancer patients and for the autonomic and rest/activity regulation measuring questionnaire.

There are several limitations in our study. The study group is heterogeneously constituted, the time-span for first diagnosis and study inclusion in particular has a high variability. Even if we have initial evidence supported by this data that self-regulation may have an influence on survival of cancer patients [19], we need more research with larger samples including sufficient male participants, that allow for every cancer type a stage adjusted analysis including detailed biological prognostic factors and therapies. Furthermore, rest/activity rhythm should be co-measured actigraphically.

## Conclusions

We have found that self-regulation might be an independent prognostic factor for the survival of breast and colon carcinoma patients. Further prospective studies with larger populations, more detailed phenotyping of patients and longer follow-up are required to confirm this finding. Ultimately we need to test methods to improve self-regulation in cancer patients as part of oncological management.

## List of abbreviations

aR: autonomic regulation; BC: breast cancer; C: control; CRC: colorectal cancer; CRF: Cancer related fatigue; HRQL: Health-related quality of life; SOC: Sense of coherence; SR: self-regulation; UICC: Union Internationale Contre le Cancer

## Competing interests

The authors declare that they have no competing interests, and were free to interpret the data according to a strict scientific rationale.

## Authors' contributions

MK, RZ, HBvL, MG initiated the project, and contributed to the project design and data collection, MR and ABo participated in the initiation of the project and performed statistical analyses, MK, MR, RZ, GF, AB, ABo, SNW, HM, MG contributed to interpretation, and MK, MR, GF, AB, ABo contributed to the writing of the paper. All authors read and approved the final manuscript.
